# 

**Published:** 1897-03

**Authors:** 


### THE BRISTOL

### Medico-Chirurgical Journal.

A JOURNAL OF THE MEDICAL SCIENCES FOR THE WEST OF ENGLAND AND SOUTH WALES

*PUBLISHED UNDER THE AUSPICES OF*

*THE BRISTOL MEDICO-CHIRURGICAL SOCIETY.*

EDITOR:

R. SHINGLETON SMITH, M.D., B.Sc.

WITH WHOM ARE ASSOCIATED

J. MICHELL CLARKE, M.A., M.D.

F. R. CROSS, M.B. AND W. H. HARSANT.

ASSISTANT-EDITOR:

L. M. GRIFFITHS.

EDITORIAL SECRETARY:

B. M. H. ROGERS, B.A., M.D., B.Ch.

"Scire est nescire, nisi id me
Scire alius sciret."

*VOL. XV.*

BRISTOL: J. W. ARROWSMITH.
London: J. & A. Churchill, 7 Great Marlborough Street.
1897.

L.H.

R 31
B 8

J. W. Arrowsmith Publisher Bristol

### CONTENTS OF VOLUME XV.

| | Page |
|---|---|
| The Effect of the Röntgen Rays on Calculi. By JAMES SWAIN, M.S., M.D. Lond., F.R.C.S. Eng. ... ... ... ... ... ... ... | 1 |
| The Symptoms and Treatment of Chronic Suppuration of the Maxillary Antrum. By BARCLAY J. BARON, M.B. Edin. ... ... | 13 |
| Secondary Rashes in Scarlet Fever. By J. O. SYMES, M.D. Lond.... | 20 |
| Three Cases of Vaginal Hysterectomy for Malignant Disease. By W. H. C. NEWNHAM, M.A., M.B. Cantab., M.R.C.S. Eng. ... ... | 24 |
| Two Cases of Compound Depressed Fracture of Frontal Bone. By ARTHUR W. PRICHARD, M.R.C.S. Eng. ... ... ... ... ... ... | 27 |
| A Case of Hysterical Disease of both Hips and both Knees, with Extreme Distortion of the Lower Limbs. By CHARLES A. MORTON, F.R.C.S. Eng. ... ... ... ... ... ... ... ... ... | 30 |
| In Memoriam: JAMES GREIG SMITH... ... ... ... ... ... ... ... ... | 105 |
| Paroxysmal Tachycardia. By P. WATSON WILLIAMS, M.D. Lond. | 122 |
| Paralysis of the Respiratory Centre from Intracranial Pressure. By J. LACY FIRTH, M.S., M.D. Lond., F.R.C.S. Eng.... ... ... ... | 139 |
| Two Cases of Abdominal Section. By J. RAGLAN THOMAS, M.D. Lond., M.R.C.S. Eng. ... ... ... ... ... ... ... ... ... ... | 145 |
| A Case of Acute Miliary Tuberculosis. By J. MICHELL CLARKE, M.A., M.D. Cantab., F.R.C.P. ... ... ... ... ... ... ... ... | 148 |
| A Case of Ruptured Tubal Pregnancy. By W. H. C. NEWNHAM, M.A., M.B. Cantab., M.R.C.S. Eng. ... ... ... ... ... ... ... | 152 |
| Fifteen Years' Experience of Infectious Diseases in a Public School. By W. JOHNSTONE FYFFE, B.A., M.D. Dub. ... ... ... ... ... | 221 |
| The Causes of Death in Five Cases in a Colliery Explosion. By TRAFFORD MITCHELL, M.D. Glasg., D.P.H. Camb. ... ... ... | 235 |

*Page*

Two Cases of Intubation in Adults. By Harold F. Mole, F.R.C.S. Eng. ... ... ... ... ... ... ... ... ... ... ... ... ... ... 237

A Case of Hæmophilia with Joint-Lesions. By J. E. Shaw, M.B. Edin. 240

Some Points in Lunacy Practice in relation to the General Practitioner. By J. E. Shaw, M.B. Edin. ... ... ... ... ... ... ... ... ... ... 301

The Morphology and Pathology of the Pharyngeal Pouch of Rathke. By Thomas Carwardine, M.S. Lond., F.R.C.S. Eng. ... ... 310

Cases of Hepatic and Intestinal Surgery. By Charles A. Morton, F.R.C.S. Eng.... ... ... ... ... ... ... ... ... .. ... ... ... 317

Progress of the Medical Sciences ... ... ... 33, 154, 246, 326

Reviews of Books... ... ... ... ... ... ... ... ... 53, 175, 260, 344

Notes on Preparations for the Sick ... ... ... 83, 203, 286, 361

Meetings of Societies ... ... ... ... ... ... ... 87, 206, 362

The Library of the Bristol Medico-Chirurgical Society ... ... ... ... ... ... ... ... ... 96, 214, 289, 367

Local Medical Notes ... ... ... ... ... ... 99, 217, 292, 379

Scraps ... ... ... ... ... ... ... ... ... ... 101, 218, 295, 383

### The Library of the Bristol Medico-Chirurgical Society.

*The following donations have been received since the publication of the List in December:*

*February 27th, 1897.*

| | |
|---|---|
| J. Michell Clarke, M.D. (1) ... ... ... ... ... | 2 volumes. |
| Carey Coombs, M.D. (2) ... ... ... ... ... | 1 volume. |
| George M. Gould, M.D. (3) ... ... ... ... ... | 43 volumes. |
| L. M. Griffiths (4) ... ... ... ... ... ... ... | 2 ,, |
| J. Greig Smith (5) ... ... ... ... ... ... ... | 76 ,, |
| R. Shingleton Smith, M.D. (6) ... ... ... ... | 3 ,, |
| The Surgeon-General, United States Army (7)... | 1 volume. |
| J. Wright and Co. (8) ... ... ... ... ... ... | 1 ,, |

Unbound periodicals have been received from Dr. George M. Gould. One Framed Picture has been presented by Mr. L. M. Griffiths, and one by Mr. Arthur W. Prichard.

### TWENTY-THIRD LIST OF BOOKS.

The titles of books mentioned in previous lists are not repeated.

The figures in brackets refer to the names of the donors, and show by whom the volumes were presented. The books to which no such figures are attached have either been bought from the Library Fund or received through the *Journal*.

| | | |
|---|---|---|
| **Allbutt and W. S. Playfair, T. C.** [Eds.] *A System of Gynæcology* ... ... | | 1896 |
| **Barrett, A. W.** ... | *Dental Surgery* ... ... ... ... ... ... ... 3rd Ed. | 1897 |
| **Bramwell, B.** ... | *Intracranial Tumours* ... ... ... ... ... ... ... ... | 1888 |
| **Brindel, —** ... ... | *Resultats de l'Examen histologique de 64 Végétations adénoïdes* ... ... ... ... ... ... ... ... | 1896 |
| **Brown, A. M.** ... | *The Animal Alkaloids* ... ... ... ... ... 2nd Ed. | 1889 |
| **Bryant, T.** ... ... | *On Tension, Inflammation of Bone, and Head Injuries* | 1888 |
| **Buck, A. H.** ... | *Medical Dictionary* ... ... ... ... ... ... ... ... ... | 1897 |
| **Butler-Smythe, A. C.** | *A First Series of Fifty-four consecutive Ovariotomies* ... | 1897 |
| **Campbell, F.** ... | *The Theory of National and International Bibliography* | 1896 |
| *Catalogue (Index) of the Library of the Surgeon-General's Office, United States Army* ... ... ... ... 2nd Series, Vol. I. (7) | | 1896 |
| **Cautley, E.** ... ... | *The Feeding of Infants* ... ... ... ... ... ... ... ... | 1897 |
| **Chaplin, Sir A. Clark, W. J. Hadley and A.** | *Fibroid Diseases of the Lung* | 1894 |
| **Cheyne, W. W.** ... | *The Treatment of Wounds, Ulcers, and Abscesses.* 2nd Ed. | 1897 |
| **Clark, W. J. Hadley and A. Chaplin, Sir A.** | *Fibroid Diseases of the Lung* | 1894 |
| **Coombs, C.** ... ... | *Galvanism in the Treatment of Neuritis, &c.* ... (2) | 1896 |
| **Dorland, W. A. N.** | *A Manual of Obstetrics* ... ... ... ... ... ... ... | 1896 |

| | | |
|---|---|---|
| **Ehlers, E.** ... ... | *Aetiologische Studien über Lepra* ... ... ... ... ... ... | 1896 |
| **Fernie, W. T.** ... | *Herbal Simples* ... ... ... ... ... ... ... 2nd Ed. | 1897 |
| **Gould and W. L. Pyle, G. M.** | *Anomalies and Curiosities of Medicine* ... | 1897 |
| **Gowers, W. R.** ... | *Clinical Lectures on Nervous Diseases* ... ... ... ... | 1895 |
| **Grawitz, E....** ... | *Klinische Pathologie des Blutes* ... ... ... ... ... ... | 1896 |
| **Gull, [Sir] W. W.** | *A Collection of Published Writings — Memoir and Addresses.* (Ed. by T. D. Acland) ... ... ... | 1896 |
| **Hadley and A. Chaplin, Sir A. Clark, W. J.** | *Fibroid Diseases of the Lung* | 1894 |
| **Hare, H. A....** ... | *Mediastinal Disease*... ... ... ... ... ... ... ... ... | 1889 |
| **Herschell, G.** ... | *Cycling as a Cause of Heart Disease* ... ... ... ... | 1896 |
| **Jacobson, W. H. A.** | *The Operations of Surgery* ... ... ... ... 3rd Ed. | 1897 |
| **Jaruntowsky, A. von** | *Sanatoria for Consumptives.* (Tr. by E. C. Beale) ... | [N.D.] |
| **Kinsey-Morgan, A.** | *The Climate of Bournemouth in relation to Disease, especially Phthisis*... ... ... ... .. ... ... | 1897 |
| **Kirstein, A....** ... | *Autoscopy of the Larynx and the Trachea.* (Tr. by M. Thorner) ... ... ... ... ... ... ... ... | 1897 |
| **Macnaughton-Jones, H.** | *Diseases of Women*... ... ... ... ... ... 7th Ed. | 1897 |
| **Maylàrd, A. E.** ... | *The Surgery of the Alimentary Canal* ... ... ... ... | 1896 |
| **Moure, E. J.** ... | *Empyème du Sinus maxillaire chez les Enfants. Des Déviations et Éperons de la Cloison du Nez chez les jeunes Enfants* ... ... ... ... ... ... ... | 1896 |
| **Murray, W.** ... | *Rough Notes on Remedies* ... ... ... ... 2nd Ed. | 1897 |
| **Murrell, W.** ... | *What to do in cases of Poisoning* ... ... ... 8th Ed. | 1897 |
| **Napier, A. D. L....** | *The Menopause and its Disorders* ... ... ... ... ... | 1897 |
| **Naunyn, B.** ... ... | *A Treatise on Cholelithiasis.* (Tr. by A. E. Garrod) ... | 1896 |
| **Nicolas, Sir H.** ... | *The Chronology of History* ... ... ... ... ... ... (4) | 1833 |
| **Playfair, T. C. Allbutt and W. S.** [Eds.] | *A System of Gynæcology* ... ... | 1896 |
| **Poten, W.** ... ... | *Die chirurgische Asepsis der Hände* ... ... ... ... | 1897 |
| **Pye, W.** ... ... | *Elementary Bandaging and Surgical Dressing.* 7th Ed. (Revised by G. B. Smith) ... ... ... ... | 1896 |
| **Pyle, G. M. Gould and W. L.** | *Anomalies and Curiosities of Medicine* ... | 1897 |
| **Ross, F. W. F.** ... | *Intestinal Intoxication in Infants* ... ... ... ... ... | 1897 |
| **Snell, E. H....** ... | *Compressed Air Illness* ... ... ... ... ... ... ... ... | 1896 |
| **Stoney, Emily A. M.** | *Practical Points in Nursing* ... ... ... ... ... ... ... | [N.D.] |
| **Thudichum, J. L. W.** | *The Progress of Medical Chemistry* ... ... ... ... | 1896 |
| **Woakes, E.** ... ... | *On Deafness, Giddiness, and Noises in the Head.* 4th Ed. | 1896 |
| **Yearsley, M.** ... | *Injuries and Diseases of the Ear* ... ... ... ... ... | 1897 |
| **Ziegler, E.** ... ... | *Special Pathological Anatomy.* 3rd Ed. Sections I.—VIII. (Tr. and Ed. by D. Mac Alister and H. W. Cattell) ... ... ... ... ... ... ... | 1896 |

---

### TRANSACTIONS, REPORTS, JOURNALS, &c

| | | |
|---|---|---|
| American Gynæcological and Obstetrical Journal, The ... | ... Vol. IX. | 1896 |
| American Gynecological Society, Transactions of the ... | Vol. XXI. | 1896 |
| American Journal of the Medical Sciences, The ... ... | Vol. CXII. | 1896 |
| American Ophthalmological Society, Transactions of the | Vol. VII., Part III. | 1897 |
| American Public Health Association, Reports and Papers presented at the Meetings of the ... ... ... ... | (3) Vols. I.—XVII. | 1875-92 |

American Surgical Association, Transactions of the (5) Vols. VII., VIII. 1889-90
Annals of Surgery ... ... Vols. I.—V., 1885-87; VIII., 1888; XV. 1892
Archives de Neurologie ... ... ... ... ... ... ... 2e Sér., Tome II. 1896
Asclepiad, The ... ... ... ... ... ... ... ... ... ... ... Vol. XI. 1895
Association of American Physicians, Transactions of the ... Vol. XI. 1896
Australasian Medical Gazette, The ... ... ... (5) Vols. IV.—VI. 1885-87
Australian Medical Journal, The ... ... (5) N.S., Vols. VI.—IX. 1884-87
Birmingham Medical Review, The ... Vols. (5) XXVI., 1889; XL. 1896
Boston Medical and Surgical Journal, The ... ... ... Vol. CXXXV. 1896
Bristol and Clifton Directory ... ... ... ... ... ... ... (4) 1890; (8) 1897
British Journal of Dermatology, The ... ... ... ... ... Vol. VIII. 1896
British Medical Journal, The ... ... ... ... ... ... Vol. II. for 1896
British Orthopædic Transactions... ... ... ... ... ... ... Vol. I. 1896
Canada Medical and Surgical Journal ... (5) Vols. XIII.—XV. 1885-87
Centralblatt für Chirurgie ... ... ... ... ... ... ... ... ... ... 1896
Centralblatt für Gynäkologie... ... ... ... ... ... ... .. ... ... 1896
Centralblatt für innere Medicin ... ... ... ... ... ... ... ... ... 1896
Cincinnati Lancet-Clinic, The Vols. (5) LIII.—LXII., 1885-89; LXXVI. 1896
College of Physicians of Philadelphia, Transactions of the (3) 3rd Ser., Vols. XI., XII. 1889-90
Dublin Journal of Medical Science, The Vols. (6) LXXXI., LXXXII., 1886; (5) LXXXV.—LXXXVII., 1888-89; CXII. 1896
Edinburgh Medical Journal ... ... Vols. (5) XXXIV., Part 1., 1888; (1) XXXV., 1890; XLII. 1896
France médicale, La ... ... ... ... ... ... ... ... ... ... ... ... 1896
Gazetta Medica Lombarda ... ... ... ... ... ... ... ... ... ... ... 1896
Gazette des Hopitaux ... ... ... ... ... ... ... ... ... ... ... ... 1896
Gazette des Hopitaux de Toulouse ... ... ... ... ... ... ... ... ... 1896
Gazette hebdomadaire des Sciences médicales de Bordeaux Tome XVII. 1896
Glasgow Medical Journal, The ... ... ... ... ... (5) Vol. XXXIII. 1890
Hazell's Annual ... ... ... ... ... ... ... ... ... ... ... ... ... ... 1897
Hospitals—
Guy's Hospital Reports ... ... ... ... .. ... Vol. LII. 1896
Iowa State Medical Society, Transactions of the ... ... Vol. XIV. 1896
Journal of Cutaneous and Genito-Urinary Diseases ... Vol. XIV. [1896]
Journal of Laryngology, Rhinology, and Otology, The ... ... Vol. XI. 1896
Journal of Nervous and Mental Disease, The ... ... ... (3) Vol. III. 1876
Journal of the American Medical Association, The Vols. (5) IV.—XII., 1885-89; XXVII. 1896
Laboratory Reports, Royal College of Physicians, Edinburgh (5) Vol. I. 1889
Lancet, The ... ... ... ... ... ... ... ... ... ... Vol. II. for 1896
Library, The ... ... ... ... ... ... ... ... ... ... Vol. VIII. 1896
Mathews' Medical Quarterly... ... ... ... ... ... ... ... Vol. III. 1896
Medical Age, The ... ... ... ... Vols. (5) V., VI., 1887-88; XIV. 1896
Medical and Surgical Reporter, The ... Vols. (3) XXXIV.—XXXVI., 1876-77; (5) LI.—LV., 1884-86, LVII.—LXI., 1887-89; LXXV. 1896
Medical Annual, The ... ... ... ... ... ... ... ... ... ... ... ... 1897
Medical Directory, The ... ... ... ... ... ... ... ... ... ... ... 1897
Medical Magazine, The ... ... ... ... ... ... ... ... (6) Vol. V. 1896

| | | |
|---|---|---|
| Medical News, The | Vol. LXIX. | 1896 |
| Medical Press and Circular, The | [Vol. CXIII.] | 1896 |
| Medical Record | Vol. L. | 1896 |
| Medical Society's Transactions, The | Vol. XIX. | 1896 |
| Medical Standard, The | (5) Vol. III. | 1888 |
| Medicinische Bibliographie | (5) | 1885–87 |
| Monthly Abstract of Medical Science, The | (3) Vols. III., IV. | 1876–77 |
| Münchener medicinische Wochenschrift | | 1896 |
| New York Medical Journal, The | Vols. (3) XXIII.—XXV., 1876–77; LXIV. | 1896 |
| New Zealand Medical Journal, The | Vol. IX. | 1896 |
| Nord médical, Le | Tome II. | 1896 |
| Nordiska Kongressen för Invärtes Medicin, Förhandlingar vid Första | | 1896 |
| Odontological Society of Great Britain, Transactions of the. | N.S, Vol. XXVIII. | 1896 |
| Ophthalmological Transactions | Vol. XVI. | 1896 |
| Pediatrics | Vol. II. | 1896 |
| Pennsylvania, Transactions of the Medical Society of | (3) Vols. XIII., Part II.—XXV. | 1881-94 |
| Philadelphia Medical Times | (3) Vols. VIII., IX. | 1878–79 |
| Practitioner, The | Vol. LVII. | 1896 |
| Progrès médical, Le | 3e Sér., Tome IV. | 1896 |
| Retrospect of Medicine, Braithwaite's | Vol. CXIV. | 1897 |
| Revue hebdomadaire de Laryngologie, d'Otologie, et de Rhinologie | Tome XVI. | 1896 |
| Saint Louis Medical and Surgical Journal, The | (5) Vols. XLVI.—XLVIII., 1884–85, LI.—LVI. | 1886–89 |
| Sperimentale, Lo | (5) Tom. LI.—LXIII. | 1883–89 |
| Union médicale, L' | 4e Sér., Tome II. | 1896 |
| West London Medical Journal | Vol. I. | 1896 |
| Whitaker's Almanack | | 1897 |
| Year-Book of Treatment, The | | 1897 |

### Local Medical Notes.

### BRISTOL.

During the past quarter of the year 1897 two events closely connected with the medical profession have taken place. The first and the one which will leave the most permanent mark is the decision to raise money in Commemoration of Her Majesty's Diamond Jubilee to found and endow a Convalescent Home in or near the city. This decision was come to at a meeting called by the Mayor; but there can be no doubt that the minds of the persons present were influenced by the statement made by the Prince of Wales that the Queen hoped that whatever form the Commemoration took the needs of the sick poor would find some place and by the resolution in favour of a Convalescent Home passed by a joint-committee of the Infirmary and Hospital.

The second event referred to was the reading—or it might be almost called a recitation—of Dickens's "Christmas Carol" by Mr. Bancroft. The only matter of regret is that the audience was not larger, but from the view of those present that was their loss. We need not criticise Mr. Bancroft's reading. He is such a well-known actor that high expectations were not unnaturally formed of the entertainment, and the audience by their applause signified that they were not only not disappointed, but that they heartily appreciated Mr. Bancroft's reading and rendering of the story of Scrooge, and his magnanimity in coming to Bristol to give his services on behalf of the Royal Infirmary. The few words spoken by the Duke of Beaufort, who occupied the chair, conveyed in a graceful manner the thanks of the audience to the reader.

University College.—Prof. Markham Skerritt has been nominated by the Royal College of Physicians of London, Bradshaw Lecturer for 1897.

The following students have recently passed examinations:

**University of London.**—*Intermediate Examination. Excluding Physiology*—(Second Division), D. T. Price, P. G. Stock; *Physiology only*—(First Division), T. Aubrey. *Final Examination for M.D. Degree. Medicine*—G. H. Barker, B.Sc. *Final Examination for B.S. Degree*—(Second Division), G. B. Price, M.B. Lond., M.R.C.S. Eng., L.R.C.P. Lond.

**Examining Board in England by the Royal Colleges of Physicians and Surgeons.**—*Five Years' Curriculum. First Examination. Part I. Chemistry and Physics*—J. C. Clayton; *Part II. Practical Pharmacy*—G. H. Irvine, J. C. Wadmore. *Second Examination. Anatomy and Physiology*—W. N. Blatchford. *Final Examination. Medicine, Surgery, and Midwifery*—A. S. Cobbledick, R. E. W. Jennings, H. F. Parsons, H. J. Thomas; *Medicine*—C. R. Dykes*; *Surgery*—W. M. Bergin, J. J. S. Lucas, H. L. W. Norrington*; *Midwifery*—J. E. Long, R. J. Rogers.

**Society of Apothecaries of London.**—*Pass List.* February, 1897. *Midwifery*—A. H. Grace.*

**Army Medical Staff.**—H. L. W. Norrington.

At Netley. in January Mr. G. E. F. Stammers's combined marks secured him the first place in the examination and gained him the Herbert Prize of £20 for general proficiency, the Montefiore Medal and Prize of 20 guineas for surgery, the Parkes Memorial Medal for hygiene, and the Pathology Prize presented by the Director-General, A.M.D.

General Hospital.—The following appointments have been made recently: Consulting Physician-Accoucheur, A. E. Aust Lawrence, M.D., C.M. Aberd.; Physician-Accoucheur, W. H. C. Newnham, M.A., M.B. Cantab.; Assistant Physician-Accoucheur, D. C. Rayner, F.R.C S. Eng., L.R.C.P. Lond.

Hospital for Sick Children and Women.—J. O. Symes, M.D. Lond., has been appointed Anæsthetist.

* Completes Examination.

### Bristol Medico-Chirurgical Society.

---

**President.**
A. E. AUST LAWRENCE, M.D., C.M. Aberd.

**President-Elect.**

J. E. SHAW, M.B., C.M. Ed.

**Honorary Secretary.**
J. PAUL BUSH.

**Committee.**

*Past Presidents.*
AUGUSTIN PRICHARD, M.D. Berlin.
J. G. SWAYNE, M.D. Lond.
E. LONG FOX, M.D. Oxon.
W. JOHNSTONE FYFFE, M.D., B.A. Dub.
F. POOLE LANSDOWN.
L. M. GRIFFITHS.
R. SHINGLETON SMITH, M.D., B.Sc. Lond.
NELSON C. DOBSON.
S. H. SWAYNE.
F. R. CROSS, M.B. Lond.
E. MARKHAM SKERRITT, M.D., B.S., B.A. Lond.
J. GREIG SMITH, M.B., C.M., M.A. Aberd., F.R.S.E.
A. J. HARRISON, M.B. Lond.
ARTHUR W. PRICHARD.

J. MICHELL CLARKE, M.D., M.A. Cantab.
JOHN DACRE.
W. H. HARSANT.
B. M. H. ROGERS, M.D., B.Ch., B.A. Oxon.
G. MUNRO SMITH.
H. WALDO, M.D., C.M. Aberd.

Medical Practitioners wishing to join the Society are requested to communicate with the Honorary Secretary, J. PAUL BUSH, Vyvyan House, Clifton Park, Clifton, Bristol. The Annual Subscription is 21/-.

---

*Extracts from Journal By-laws.*

4.—The *Journal* shall be delivered free to Subscribers and also to Members of the Society whose subscriptions are not in arrear.

6.—The Annual Subscription to the *Journal* for those who pay before the 1st of July shall be 5/-, post free, or 6/- if paid after that date.

---

Communications intended for insertion in the *Journal* should be sent to the Editor, Dr. SHINGLETON SMITH, Deepholm, Clifton Park, Clifton, Bristol.

Books for Review and Exchange Journals should be sent to the Assistant-Editor, L. M. GRIFFITHS, 9 Gordon Road, Clifton, Bristol.

Communications referring to the delivery of the *Journal* should be sent to the Editorial Secretary, Dr. BERTRAM ROGERS, 11 York Place, Clifton, Bristol, to whom Subscriptions are to be paid *in advance*.

Cases for Binding Vols. I.–XIV. are now ready, and may be obtained, price 7*d*. each (postage 2*d*. extra), upon application to J. W. ARROWSMITH, Quay Street, Bristol; or the *Journal* will be bound, including Case, for 1/3 per volume.

### Bristol Medico-Chirurgical Society.

---

### PAST PRESIDENTS.

1874—75. FREDERICK BRITTAN, M.D., B.A. Dub.

1875—76. ROBERT WILLIAM COE.

1876—77. SAMUEL MARTYN, M.D. Ed.

1877—78. AUGUSTIN PRICHARD, M.D. Berlin.

1878—79. HENRY EDWARD FRIPP, M.D. Würzburg.

1879—80. WILLIAM MICHELL CLARKE.

1880—81. JOSEPH GRIFFITHS SWAYNE, M.D. Lond.

1881—82. EDWARD LONG FOX, M.D. Oxon.

1882—83. JAMES GEORGE DAVEY, M.D. St. And.

1883—84. WILLIAM JOHNSTONE FYFFE, M.D., B.A. Dub.

1884—85. GEORGE FORSTER BURDER, M.D. Aberd.

1885—86. WILLIAM HENRY SPENCER, M.D., M.A. Cantab.

1886—87. FRANCIS POOLE LANSDOWN.

1887—88. LEMUEL MATTHEWS GRIFFITHS.

1888—89. ROBERT SHINGLETON SMITH, M.D., B.Sc. Lond.

1889—90. NELSON CONGREVE DOBSON.

1890—91. SAMUEL HENRY SWAYNE.

1891—92. FRANCIS RICHARDSON CROSS, M.B. Lond.

1892—93. EDWARD MARKHAM SKERRITT, M.D., B.S., B.A. Lond.

1893—94. JAMES GREIG SMITH, M.B., C.M., M.A. Aberd., F.R.S.E.

1894—95. ALFRED JAMES HARRISON, M.B. Lond.

1895—96. ARTHUR WILLIAM PRICHARD.

1897.

---

### LIST OF SUBSCRIBERS

TO THE

### Bristol Medico-Chirurgical Journal.

---

*Those marked * are Members of the Society.*

| | |
|---|---|
| Ackland, J. McKno ... ... ... ... ... ... | 24 Southernhay, Exeter. |
| *Ackland, W. R. ... ... ... ... ... ... ... | 5 Rodney Place, Clifton. |
| Adams, George ... ... ... ... ... ... ... | 1 Imperial Road, Redland. |
| Adye, W. J. A. ... ... ... ... ... ... ... ... | Church House, Bradford, Wilts. |
| Aldridge, Charles, M.D., C.M. Aberd.... ... | Plympton. |
| Aldous, George F.... ... ... ... ... ... ... | Charlton House, Mannamead, Plymouth. |
| Alexander, James, M.D., M.Ch., B.A. Dub.... | Bishop's Place, Paignton. |
| Ambrose, J., M.B., C.M. Aberd. ... ... ... ... | 5 Heath Villas, Eastville, Bristol. |
| *Atchley, Cuthbert ... ... ... ... ... ... | 66 Cotham Road, Bristol. |
| Avarne, A. B. ... ... ... ... ... ... ... ... | Blaenavon, Monmouthshire. |
| Aveline, H. T. S. ... ... ... ... ... ... ... | Somerset Asylum, Taunton. |
| | |
| *Ballance, H. Stanley, M.D. Lond. ... ... | 4 Royal Terrace, Weston-super-Mare. |
| Ballantyne, J. W., M.D., C.M. Edin. ... ... | 24 Melville Street, Edinburgh. |
| Bannatyne, Gilbert A., M.D., C.M. Glasg.... | 1 Paragon, Bath. |
| *Barclay, W. M. ... ... ... ... ... ... ... | Queen's Road, Clifton. |
| Baretti, T. G. L'Enardi ... ... ... ... ... | 49 Royal York Crescent, Clifton. |
| *Baron, Barclay J., M.B., C.M. Ed.... ... ... | 16 Whiteladies Road, Clifton. |
| Barr, J. Coleman ... ... ... ... ... ... ... | 2 Cranmore Cottages, Aldershot. |
| Barrett, W. F. H. ... ... ... ... ... ... ... | Ottery St. Mary, Devonshire. |
| *Basset, Walter ... ... ... ... ... ... ... | 24 Stow Hill, Newport, Mon. |
| Batten, Rayner W., M.D. Lond. ... ... ... | 1 Brunswick Square, Gloucester. |
| Batten, W. Smith... ... ... ... ... ... ... | Curzon House, Calne. |
| *Beaver, H. Atwood, M.B., B.Ch.Vict. ... ... | 6 Downleaze Road, Stoke Bishop. |
| *Beddoe, John, M.D.Ed., B.A. Lond., F.R.S. | The Chantry, Bradford-on-Avon. |
| Benham, Harry A., M.D., C.M. Aberd. ... ... | Asylum, Stapleton, Gloucestershire. |
| Bennett, W. S. ... ... ... ... ... ... ... | Escot, Penzance. |
| *Benson, A. H. ... ... ... ... ... ... ... ... | 66 Barton Street, Gloucester. |
| *Berry, F. C., M.D., B.Ch., B.A. Dub. ... ... | Burnham, Somersetshire. |

### LIST OF SUBSCRIBERS.

| | |
|---|---|
| Berry, James, M.B., B.S. Lond. ... ... ... | 60 Welbeck Street, London, W. |
| Bevan, W. L. P., M.D., C.M. Ed. ... ... ... | Alton, Hampshire. |
| Biamonti, Sebastiano ... ... ... ... ... ... | 27 Via Balbi, Genoa, Italy. |
| *Blacker, A. E., M.D. Dub. ... ... ... ... ... | Richmond Hill Avenue, Clifton. |
| *Blagg, Arthur F. ... ... ... ... ... ... ... | 6 West Mall, Clifton. |
| Blomfield, Arthur G., M.D. Aberd. ... ... | 34 West Southernhay, Exeter. |
| *Board, Edmund C. ... ... ... ... ... ... ... | 11 Caledonia Place, Clifton. |
| Boyce, James G. ... ... ... ... ... ... ... | The Chipping, Wotton-under-Edge. |
| *Brasher, Charles W. J. ... ... ... ... ... | 128 Cotham Brow, Bristol. |
| Brimacombe, R. William ... ... ... ... ... | Kingswood, Bristol. |
| *Broom, John, M.D. Brux. ... ... ... ... ... | St. Paul's Road, Clifton. |
| Brough, C. A. La T., M.B., B.S., LL.D. Durh. | University College, Bristol. |
| Brown, G. Arthur ... ... ... ... ... ... | Tredegar. |
| Brown, William, M.D., C.M. Glasg. ... ... | Fishponds, Gloucestershire. |
| Browne, J. Walton, M.D., B.A. Royal Univ. I. | 10 College Square North, Belfast. |
| *Bullen, F. St. John ... ... ... ... ... ... | 12 Pembroke Road, Clifton. |
| Burroughs, E. F. H. ... ... ... ... ... ... | 3 Elgin Park, Bristol. |
| *Bush, J. Paul ... ... ... ... ... ... ... ... | Clifton Park, Clifton. |
| | |
| Campbell, George ... ... ... ... ... ... | Chilton Polden, Somersetshire. |
| *Capron, H. J. ... ... ... ... ... ... ... ... | Princes Road, Clevedon. |
| *Carr, Alexander ... ... ... ... ... ... ... | 150 Wells Road, Bristol. |
| *Carter, T. M. ... ... ... ... ... ... ... ... | 3 Clifton Vale, Clifton. |
| *Carter, W. F. ... ... ... ... ... ... ... ... | 48 Coronation Road, Bristol. |
| *Carwardine, Thomas, M.B., M.S. Lond. ... | Royal Infirmary, Bristol. |
| Cave, Edward J., M.D. Lond. ... ... ... ... | Crewkerne. |
| Cheves, Alexander B., M.D., M.A. Aberd. ... | Millbrook, Devonshire. |
| *Chilton, R. H. ... ... ... ... ... ... ... | North Point, Durdham Park, Bristol. |
| Church, Basil... ... ... ... ... ... ... ... | Minchinhampton, Gloucestershire. |
| *Clarke, John Michell, M.D., M.A. Cantab. | 28 Pembroke Road, Clifton. |
| Clay, Challoner ... ... ... ... ... ... ... | Manor House, Fovant. |
| Clay, R. Hogarth, M.D. Ed. ... ... ... ... | 4 Windsor Villas, Plymouth. |
| Coates, C. J. A. ... ... ... ... ... ... ... | Chatteris, Cambridgeshire. |
| Cobbold, C. S. W., M.D. Würzburg... ... ... | Bailbrook House, Bath. |
| Coker, T. V. ... ... ... ... ... ... ... ... | 2 Chesterfield Place, Clifton. |
| Cole, Thomas, M.D. Lond. ... ... ... ... ... | 18 Circus, Bath. |
| Collins, H. W. ... ... ... ... ... ... ... | Wrington. |
| Colmer, Ptolemy S. H., M.D. Dur. ... ... | South Street House, Yeovil. |
| *Cook, Henri, M.D., C.M. Aberd. ... ... ... | 45 Stackpool Road, Bristol. |
| Cooke, A. S. ... ... ... ... ... ... ... ... | Badbrook House, Stroud. |
| Cory, W. Howard ... ... ... ... ... ... ... | 1 The Avenue, Redland. |
| Cossham, W. R., M.D., C.M. Aberd.... ... ... | Cirencester. |
| Cotton, William, M.B., C.M., M.A. Ed. ... | 227 Gloucester Road, Bristol. |
| *Coulter, R. J., M.B. Dub. ... ... ... ... ... | Eye Hospital, Bristol. |
| Cousins, J. Ward, M.D. Lond. ... ... ... ... | Kent Road, Southsea. |
| Cowan, F. S. ... ... ... ... ... ... ... ... | 27 Queen Square, Bath. |
| Craddock, Samuel... ... ... ... ... ... ... | South Lyncombe, Bath. |
| Crallan, G. E., M.A., M.B. Cantab.... ... ... | Brislington House, Brislington. |
| Crespi, Alfred J. H. ... ... ... ... ... ... | Poole Road, Wimborne. |
| *Cross, F. Richardson, M.B. Lond.... ... ... | Worcester House, Clifton. |
| *Crossman, Edward, M.D. Dur.... ... ... ... | Hambrook, Gloucestershire. |
| *Crossman, F. W. ... ... ... ... ... ... ... | Hambrook, Gloucestershire. |
| Crouch, C. Percival, M.B. Lond. ... ... ... | 3 Princes Buildings, Weston-super-Mare. |
| Culross, James, M.B., C.M., M.A. Glasg. ... | 66 Queen Street, Newton Abbot. |
| Cunningham, C. L.... ... ... ... ... ... ... | Chudleigh. |
| | |
| *Dacre, John ... ... ... ... ... ... ... ... | 14 Eaton Crescent, Clifton. |
| Dalby, Augustus W. ... ... ... ... ... ... | Redland House, Frome. |

| | |
|---|---|
| *Davies, D. S., M.D. Lond., D.P.H. Cantab. ... | 60 Oakfield Road, Clifton. |
| Davies, Evan ... ... ... ... ... ... ... ... | Maesteg, Glamorganshire. |
| Davies, H. N. ... ... ... ... ... ... ... ... | Porth, Glamorganshire. |
| Davis, Theodore, M.D. Lond. ... ... ... ... | Beachcroft, Clevedon. |
| Davy, Henry, M.D. Lond. ... ... ... ... ... | Southernhay House, Exeter. |
| Day, Percy Howard ... ... ... ... ... ... | Stalmine, Poulton-le-Fylde. |
| Dening, Edwin ... ... ... ... ... ... ... | Manor House, Stow-on-the-Wold. |
| Devis, Harry F. ... ... ... ... ... ... ... | Wells Road, Bristol. |
| Dickie, James Steel, M.D., C.M. Aberd. ... | Boscombe Park, Bournemouth. |
| *Dobson, Nelson C. ... ... ... ... ... ... | 27 Victoria Square, Clifton. |
| Domville, Edward J. ... ... ... ... ... ... | 44 Southernhay, Exeter. |
| Dunbar, Eliza L. Walker, M.D. Zürich ... | 9 Oakfield Road, Clifton. |

| | |
|---|---|
| Eadon, W. F. Bailey ... ... ... ... ... ... | Hambrook, Gloucestershire. |
| *Eager, Reginald, M.D. Lond. ... ... ... ... | Northwoods, Winterbourne. |
| Eberle, E., M.A., M.B., B.Ch. Dub. ... ... | Napier Road, Redland. |
| Eddowes, Charles ... ... ... ... ... ... | Maddington, Devizes. |
| *Edgeworth, F. H., M.B., B.C., B.A. Cantab., B.Sc. Lond. ... ... ... ... ... ... ... ... | 35 Pembroke Road, Clifton. |
| Edwards, W. T., M.D. Lond. ... ... ... ... | 129 Queen Street, Cardiff. |
| Elder, William ... ... ... ... ... ... ... ... | 7 Trelawney Road, Bristol. |
| *Elliott, Christopher, M.D., B.A. Dub. ... | 88 Pembroke Road, Clifton. |
| Ellis, W. Mc D., M.D. Brux. ... ... ... ... | 9 Bladud Buildings, Bath. |
| Elsworth, R. G., M.B., C.M. Edin.... ... ... | 203 St. Helens Road, Swansea. |
| Embleton, Dennis C. ... ... ... ... ... ... | St. Michael's Road, Bournemouth. |
| Essex, J. R. ... ... ... ... ... ... ... ... | Pontypool, Monmouthshire. |
| Evans, J. Fenton, M.B., C.M. Ed. ... ... ... | 6 Whitehall Yard, London, S.W. |
| Evans, T. G. C. ... ... ... ... ... ... ... | Budleigh Salterton, Devon. |
| *Ewens, John ... ... ... ... ... ... ... ... | The Elms, Cotham Hill, Bristol. |

| | |
|---|---|
| Fairbanks, W., M.D., C.M. Ed. ... ... ... ... | Wells, Somersetshire. |
| *Fawcett, E., M.B., C.M. Ed. ... ... ... ... | 141 Redland Road, Bristol. |
| *Fendick, R. George ... ... ... ... ... ... | St. Paul's Road, Clifton. |
| Fenwick, Charles... ... ... ... ... ... ... | Dunsford, Exeter. |
| Finch, Thomas, M.B., B.A. Cantab.... ... ... | Watcombe View, Babbacombe. |
| *Firth, J. L., M.D., B.S. Lond. ... ... ... ... | 20 Richmond Park Road, Clifton. |
| *Fisher, Theodore, M.D. Lond.... ... ... ... | 25 Pembroke Road, Clifton. |
| *Flemming, C. E. S. ... ... ... ... ... ... | Freshford. |
| Ford, Joseph ... ... ... ... ... ... ... ... | Wedmore. |
| Forty, D. H. ... ... ... ... ... ... ... ... | Wotton-under-Edge. |
| Fowler; O. H. ... ... ... ... ... ... ... ... | Cirencester. |
| Fox, Arthur E. W., M.B., C.M. Ed. ... ... | 16 Gay Street, Bath. |
| *Fox, Bonville B., M.D., M.A. Oxon. ... ... | Brislington. |
| *Fox, E. Long, M.D. Oxon. ... ... ... ... ... | Church House, Clifton. |
| Fox, John Alfred ... ... ... ... ... ... ... | Tregea House, Penzance. |
| Fox, T. Colcott, M.B. Lond., B.A. Cantab.... | 14 Harley Street, London, W. |
| Fraser, Græme B.... ... ... ... ... ... ... | Beach Road, Weston-super-Mare. |
| *Freeman, Henry W. ... ... ... ... ... ... | 24 Circus, Bath. |
| *Freeman, J. ... ... ... ... ... ... ... ... | Cheltenham Road, Bristol. |
| Frood, James N. ... ... ... ... ... ... ... | Topsham, Devon. |
| *Fyffe, W. Johnstone, M.D., B.A. Dub.... ... | 2 Rodney Place, Clifton. |
| Gamble, C. H. ... ... ... ... ... ... ... ... | Barnstaple. |
| Garland, E. B., M.B., C.M. Edin. ... ... ... | 92 Hampton Road, Redland. |
| *Garner, John E., M.D., C.M. Aberd. ... ... | 6 Wenckley Square, Preston, Lancashire. |
| Genge, T. T. ... ... ... ... ... ... ... ... | 21 Whiteladies Road, Bristol. |
| Gent, W. C. ... ... ... ... ... ... ... ... | 170 Wells Road, Bristol. |
| *Gerrish, D. G.... ... ... ... ... ... ... ... | Royal Infirmary, Bristol. |

### LIST OF SUBSCRIBERS.

| | |
|---|---|
| *Gibbs, Alfred N. Godby | 52 Whiteladies Road, Clifton. |
| Gidley, G. G. | Cullompton, Devon. |
| Goodfellow, W. R. | Roche, Cornwall. |
| Goodridge, Henry F. A., M.D. Lond. | 10 Brock Street, Bath. |
| Goodwyn, A. H. | Droxford, Hampshire. |
| Goss, T. Biddulph | 1 Circus, Bath. |
| Grace, Alfred | Chipping Sodbury. |
| Grace, Edward Mills | Thornbury, Gloucestershire. |
| Grace, W. G. | Ashley Grange, Ashley Down, Bristol. |
| Gratte, C. Brooke | 3 Lansdown Place, Newport, Mon. |
| *Gray, A. Murray | 17 Market Place, Devizes. |
| Greenway, A. G., M.D. R.U.I. | Llandrindod Wells, Radnorshire. |
| Grey, T. C. | Church House, St. Neot's. |
| *Griffiths, J. S. | 25 Redland Park, Bristol. |
| *Griffiths, L. M. | 9 Gordon Road, Clifton. |
| Griffiths, P. Rhys, M.B., B.S. Lond. | 71 Newport Road, Cardiff. |
| Groves, J., M.B., B.A. Lond. | Carisbrooke. |

| | |
|---|---|
| Hadwen, W. R. | Highbridge, Somersetshire. |
| *Hailes, Clements D. G., M.D., C.M. Ed. | 27 Alma Road, Clifton. |
| *Haines, A. H. | 8 Cotham Road, Bristol. |
| Hale, Edmund T. | The Grange, Chew Magna. |
| Halliwell, T. O. | Blagdon, Somerset. |
| Halpin, W. C. | Parkend, Gloucestershire. |
| Hamlen-Williams, T. R. | Verlands, Cowbridge, Glamorganshire |
| Handfield-Jones, Montagu, M.D. Lond. | 35 Cavendish Square, London, W. |
| Hardwick, A., M.B. Dur. | Newquay, Cornwall. |
| Harper, Joseph | Bear Street, Barnstaple. |
| *Harrison, A. J., M.B. Lond. | Guthrie Road, Clifton. |
| Harrison, Charles | Keynsham, Somersetshire. |
| Harsant, J. G., M.D., B.S. Lond. | Exeter Road, Bournemouth. |
| *Harsant, W. H. | Tower House, Clifton. |
| *Harvey, Alfred, M.B. Lond. | Westbury-on-Trym. |
| Hawkins-Ambler, G. A. | 162 Upper Parliament Street, Liverpool. |
| Hawkins, Cæsar F. | North Petherton. |
| Hayman, Alfred G. | 1 Pembroke Road, Clifton. |
| *Hayman, C. A., M.D. St. And. | Kingston Villa, Richmond Hill, Clifton. |
| *Heaven, John C., D.Ph. Lond. | 17 Whiteladies Road, Bristol. |
| Hemming, J. Hughes | Kimbolton, St. Neot's. |
| *Herapath, C. K. C. | Cotham Park, Bristol. |
| *Hetling, H. E. | 30 Elliston Road, Bristol. |
| Hill, Hedley | 1 Redcliff Hill, Bristol. |
| *Hill, Thomas, M.D. St. And. | 138 Cromwell Road, Bristol. |
| *Hill, Walter J. | Edgcumbe Villa, Clevedon. |
| Hoffman, A. W. W. | 30 The Parade, Leamington. |
| Hollings, Edwin T. | Woolacombe, Morthoe. |
| Horsfall, John, M.A. Oxon. | Richmond Hill, Bournemouth. |
| Hospital, Bristol General (*Sec.*, W. Thwaites). | |
| Hospital, Taunton and Somerset (*House-Surgeon*, W. H. Mardlow, M.D.) | |
| Houghton, Leonard F. | East Looe, Cornwall. |
| Howells, William, M.B., C.M. Glasg. | Church House, Talgarth, Breconshire. |
| Howse, William | 8 London Street, Swindon, Wiltshire. |
| Howsin, E. Arthur, M.D. St. And. | Stroud. |
| Hudson, F. Horace | Sandringham House, Dawlish. |
| Huggard, W. R., M.D., M.Ch., M.A. Roy. Univ. I. | Davos-Platz, Switzerland. |
| Humphreys, Henry, M.D., M.A. Cantab. | St. Mary Church Road, Torquay. |
| Hunt, A. D. | Millbrook House, Chagford. |
| Hyatt, J. T. | Shepton Mallet. |

### LIST OF SUBSCRIBERS.

IMLAY, G. A., M.B., C.M. Aberd. ... ... ... 1 Cotham Park, Bristol.
INSTITUTION, LIVERPOOL MEDICAL (*Resident Librarian*, WILLIAM JONES).
IVES, WILLIAM R. Y. ... ... ... ... ... ... Portswood Road, Southampton.

JACKSON, MARK, M.D. Roy. Univ. I. ... ... ... Bear Street, Barnstaple.
JACOBSON, W. H. A., M.B., M.C., M.A. Oxon. 66 Great Cumberland Place, London, W.
JAMISON, A., M.B., B.Ch., M.A. Roy. Univ. I. ... 66 Woodstock Road, Belfast.
JESSOP, CHARLES MOORE ... ... ... ... ... Clare Lodge, Redhill.
JONES, EVAN ... ... ... ... ... ... ... ... Ty-mawr, Aberdare.
JONES, H. MACNAUGHTON, M.D., M.Ch. Roy. U.I. 141 Harley Street, London, W.
JONES, W. W., M.B., C.M. Ed. ... ... ... ... 47 Wellington Street, Merthyr Tydvil.

*KEMM, F. ST. J.... ... ... ... ... ... ... ... Worle.

KENDALL, BERNARD C.... ... ... ... ... ... Elm Grove, Penally, Pembrokeshire.

*KENDALL, H. W. ... ... ... ... ... ... ... 9 King's Parade, Clifton.

KENNEDY, W. P., M.D., B.Ch., B.A. Dub. ... High Street, Lydney.

*KING, PRESTON, M.D. Cantab. ... ... ... ... 29 Gay Street, Bath.

KINNEIR, R. ... ... ... ... ... ... ... ... The Tower House, Malmesbury.

*LACE, F. ... ... ... ... ... ... ... ... ... 5 Paragon, Bath.
LANCASTER, E. LE CRONIER, B.A., M.B., B.Ch. Oxon. ... ... ... ... ... ... ... ... ... Winchester House, Swansea.
LANGRIDGE, F. W. ... ... ... ... ... ... ... Croftside, Ilfracombe.
*LANSDOWN, F. POOLE ... ... ... ... ... ... 19 Whiteladies Road, Clifton.
*LANSDOWN, R. G. P., M.B., B.S. Dur. ... ... 39 Oakfield Road, Clifton.
LAUWERS, DR. ... ... ... ... ... ... ... ... Courtrai, Belgium.
*LAWRENCE, A. E. AUST, M.D., C.M. Aberd. ... 19 Richmond Hill, Clifton.
LAWRIE, J. MACPHERSON, M.D., C.M. Glasg.... Greenhill, Weymouth.
LEACH, J. COMYNS, M.D. Dur., B.Sc. Lond. ... Sturminster Newton.
*LEES, E. LEONARD, M.D., C.M. Ed. ... ... ... Redland Road, Bristol.
LEIGH, W. W. ... ... ... ... ... ... ... ... Treharris, Glamorganshire.
LIDIARD, S. A. ... ... ... ... ... ... ... ... Kimberley Road, Falmouth.
*LIGERTWOOD, WALTER ... ... ... ... ... ... Altona, Elmdale Road, Clifton.
LINDSAY, JAMES A., M.D., M.Ch., M.A. Roy. U.I. 37 Victoria Place, Belfast.
LISTER, LORD, P.R.S. ... ... ... ... ... ... 12 Park Crescent, London, N.W.
*LOWE, T. PAGAN ... ... ... ... ... ... ... 16 Circus, Bath.

*McCREA, P. W., M.D., B.Ch., B.A., B.A.O., B.A. Dub. ... ... ... ... ... ... ... ... 19 Portland Square, Bristol.
MACDONALD, J. A., M.D., M.Ch. Roy. Univ. I. 19 East Street, Taunton.
MAC DONALD, P. W., M.D., C.M. Aberd.... ... County Asylum, Dorchester.
MAC DOUGALL, HENRY R. ... ... ... ... ... Shepscombe, Stroud.
McELFATRICK, JOHN ... ... ... ... ... ... The Chantry, Mere, Wiltshire.
MAC ILWAINE, S. W. ... ... ... ... ... ... South Petherton, Somersetshire.
MACKIE, J., M.B., LL.D. Aberd., C.M.G. ... Alexandria, Egypt.
MACKINNON, CHARLES, M.B., C.M., M.A. Glasg. Dyer Street, Cirencester.
MACLEAN, EWEN J., M.D., C.M. Ed.... ... ... 51 Linden Gardens, London, W.
MACLEAN, J. CAMPBELL, M.B., C.M. Ed.... ... Swindon House, Swindon, Wiltshire
MACREADY, J. ... ... ... ... ... ... ... ... 51 Queen Ann Street, London, W.
MARSH, O. E. BULWER ... ... ... ... ... ... Parkdale, Newport, Mon.
MARSHALL, ARTHUR L., M.B., B.A. Cantab. ... 2 Clapton Common, London.
*MARSHALL, HENRY, M.D. Ed., F.R.S.E.... ... 28 Caledonia Place, Clifton.

### LIST OF SUBSCRIBERS.

| | |
|---|---|
| *Martin, E. F., M.B. Ed. ... ... ... ... ... | Victoria House, Weston-super-Mare. |
| Mason, S. B. ... ... ... ... ... ... ... ... | 12 Park Terrace, Pontypool. |
| Mason, William ... ... ... ... ... ... ... | Sedgemoor Cottage, St. Austell. |
| *Matthews, Charles E., M.D. Dur. ... ... | 51 Pembroke Road, Clifton. |
| Mayor, T. O. ... ... ... ... ... ... ... ... | 40 Apsley Road, Clifton. |
| Meredith, John, M.D. Ed. ... ... ... ... ... | Wellington, Somersetshire. |
| *Millard, W. J. Kelson, M.D. St. And. ... ... | 2 Tweeddale Terrace, Tonbridge Wells. |
| *Mole, Harold F. ... ... ... ... ... ... ... | Royal Infirmary, Bristol. |
| Monckton, William ... ... ... ... ... ... | Portishead. |
| Morgan, Albert T., M.D. Brux. ... ... ... | 89 Chesterfield Road, St. Andrew's Park, Bristol. |
| Morgan, John ... ... ... ... ... ... ... ... | Langport, Somersetshire. |
| *Morgan, T. Whitworth S. ... ... ... ... | Pill. |
| *Morton, C. A. ... ... ... ... ... ... ... ... | 14 Vyvyan Terrace, Clifton. |
| Mouat-Biggs, C. E. F.... ... ... ... ... ... | 2 Fauconberg Villas, Cheltenham. |
| Munden, Charles ... ... ... ... ... ... ... | Ilminster. |
| Murray, William, M.B., C.M. Ed. ... ... ... | Staple Hill, Gloucestershire. |
| *Myles, G. T. ... ... ... ... ... ... ... ... | 3 Portland Square, Bristol. |

| | |
|---|---|
| *Neale, A. E., M.B., B.S. Dur. ... ... ... ... | Nevil Road, Bishopston. |
| Nell, Richard F. ... ... ... ... ... ... ... | 33 Windsor Terrace, Penarth. |
| *Nevill, W. N., M.D., B.Ch., B.A. Dub. ... ... | Southville, Bristol. |
| *Newman, Charles... ... ... ... ... ... ... | 156 Cheltenham Road, Bristol. |
| *Newnham, W. H. C., M.B., M.A. Cantab. ... | Chandos Villas, Clifton. |
| Nicholson, T. D., C.M. Ed. ... ... ... ... | 2 Whiteladies Road, Clifton. |
| Norman, J. H. ... ... ... ... ... ... ... ... | Goodleigh, Winkleigh, Devonshire. |

| | |
|---|---|
| O'Brien, Cornelius ... ... ... ... ... ... | Down House, Whitehall, Bristol. |
| Odell, William ... ... ... ... ... ... ... | Ferndale, Torquay. |
| *Ogilvy, Alexander, M.D., B.Ch., B.A. Dub. | Montrose House, Queen's Road, Clifton. |
| Openshaw, E. Hyde ... ... ... ... ... ... | Barrules, Cheddar. |
| *Ormerod, H. Lawrence, M.B., B.Ch., B.A.O., R.U.I. ... ... ... ... ... ... ... ... ... ... | Foxstones, Westbury-on-Trym. |

| | |
|---|---|
| Page, G. Shepley ... ... ... ... ... ... ... | 78 Old Market Street, Bristol. |
| *Parker, George, M.D., M.A. Cantab. ... ... | 14 Pembroke Road, Clifton. |
| Parry, J. H. ... ... ... ... ... ... ... ... | 199 Cheltenham Road, Bristol. |
| Parsons, Francis J. C.... ... ... ... ... ... | King Square, Bridgwater. |
| *Parsons, J. St. John G. ... ... ... ... ... | 6 Hillside, Cotham. |
| Paterson, Donald Rose, M.D., C.M. Ed. ... | 18 Windsor Place, Cardiff. |
| Peake, Arthur W.... ... ... ... ... ... ... | 1 Bridewell Street, Bristol. |
| Peake, G. Arthur ... ... ... ... ... ... ... | Rodney Place, Cheltenham. |
| *Penny, W. J. ... ... ... ... ... ... ... ... | Coombe, West Crewkerne, Somerset. |
| *Perry, W. A. ... ... ... ... ... ... ... ... | 13 Dowry Square, Bristol. |
| Phillips, Edward Picton ... ... ... ... ... | High Street, Haverfordwest. |
| Phillips, J. A. ... ... ... ... ... ... ... ... | 72 Carlisle Street, Cardiff. |
| Phillips, Sutherland Rees, M.D., M.Ch. Roy. Univ. I.... ... ... ... ... ... ... ... | St. Anne's Heath, Virginia Water, Surrey. |
| *Pickering, C. F. ... ... ... ... ... ... ... | 12 Berkeley Square, Bristol. |
| Plaister, W. H. ... ... ... ... ... ... ... | High Road, Tottenham. |
| *Pocock, H. I. ... ... ... ... ... ... ... ... | The Barracks, Horfield. |
| Pollard, Reginald, M.B. Dur. ... ... ... | Southlands, Torquay. |
| *Pountney, W. E., M.D., C.M. Ed. ... ... ... | 33a Whiteladies Road, Bristol. |
| Powell, J. J., M.D. Lond. ... ... ... ... ... | 2 Portland Square, Bristol. |

### LIST OF SUBSCRIBERS.

| | |
|---|---|
| POWELL, WILLIAM, M.B. Lond. ... ... ... ... | Hill Garden, Torquay. |
| PRICE, WILLIAM, M.B. Lond. ... ... ... ... | 40 Park Place, Cardiff. |
| *PRICHARD, ARTHUR W. ... ... ... ... ... ... | 6 Rodney Place, Clifton. |
| *PRICHARD, AUGUSTIN, M.D. Berlin ... ... ... | 4 Chesterfield Place, Clifton. |
| *PRICHARD, J. E., M.B., B.A. Oxon. ... ... ... | 243 Cheltenham Road, Bristol. |
| PRING, A., B.A. Cantab. ... ... ... ... ... | Clyde Road, Redland. |
| *PROWSE, ARTHUR B., M.D. Lond. ... ... ... | 5 Lansdown Place, Clifton. |
| PULLIN, THOS. H. S., M.D. ... ... ... ... ... | Sidmouth. |
| PURDY, J. R., M.D. Aber. ... ... ... ... ... | Lower Hutt, New Zealand. |

| | |
|---|---|
| *RANDALL, MARTIN, M.D. Lond. ... ... ... ... | General Hospital, Bristol. |
| RANDOLPH, CHARLES ... ... ... ... ... ... | St. Michael's, Milverton, Somersetshire. |
| *RATTRAY, J. M., M.D., C.M., M.A. Aberd. ... | Frome. |
| REDWOOD, T. HALL, M.D., C.M., M.A. Dur. ... | The Lawn, Rhymney, Monmouthshire. |
| REES, ALFRED ... ... ... ... ... ... ... ... | 46 London Square, Cardiff. |
| RENDALL, JOHN ... ... ... ... ... ... ... | Forestside, Lymington. |
| *RICHARDSON, W., M.D. St. And. ... ... ... | 1 Belgrave Villas, Cotham Grove, Bristol. |
| RIDDELL, ANDREW W. ... ... ... ... ... ... | Hurstbourne Tennant, Andover. |
| *RIGG, S. E. ... ... .. ... ... ... ... ... | United Hospitals, Bath. |
| ROBATHAN, G. B. ... ... ... ... ... ... ... | The Grove, Risca. |
| ROBERTS, FREDERICK T., M.D., B.Sc. Lond. | 102 Hartley Street, London, W. |
| RODERICK, SYDNEY J., M.B., C.M. Ed. ... ... | Llanelly. |
| RODGERS, JOHN W., M.B., C.M. Ed. ... ... | Redfield, Gloucestershire. |
| *ROGERS, B. M. H., M.D., B.Ch., B.A. Oxon. ... | 11 York Place, Clifton. |
| *ROSSITER, GEORGE F., M.B. Lond. ... ... | Cairo Lodge, Weston-super-Mare. |
| ROUÉ, W. BARRETT, M.D., M.S. Dur. ... ... | 2 Buckingham Place, Clifton. |
| ROUTH, R. HENRY F. ... ... ... ... ... ... | Bridgewater. |
| ROW, F. EVERARD ... ... ... ... ... ... ... | 9 Tamar Terrace, Devonport. |
| *ROXBURGH, ROBERT, M.B. Ed. ... ... ... | 1 Victoria Buildings, Weston-super-Mare. |
| *RUDGE, C. KING ... ... ... ... ... ... ... | 145 Whiteladies Road, Bristol. |
| *RUDGE, H. T. ... ... ... ... ... ... ... ... | 5 Colston Parade, Bristol. |
| RUFFER, M. ARMAND, M.D., B.S., M.B. Oxon. | Cairo, Egypt. |
| RUTHERFORD, H. T. ... ... ... ... ... ... | Park Street, Taunton. |

| | |
|---|---|
| SCOTT, JAMES, M.B., C.M. Ed. ... ... ... ... | 24 Gaolgate Street, Stafford. |
| SCOTT, RICHARD, J. H. ... ... ... ... ... ... | 28 Circus, Bath. |
| SEARLE, GEORGE C. ... ... ... ... ... ... | Brixham, Devonshire. |
| SEARLE, RICHARD BURFORD... ... ... .. ... | Bexley, Dartmouth. |
| SEMPLE, H. F. ... ... ... ... ... ... ... ... | Budleigh Salterton, Devonshire. |
| SEMPLE, W. R. M., M.B., C.M. Glasg. ... ... | Henford Park, Yeovil, Somersetshire. |
| SEWARD, W. J., M.B. Lond. ... ... ... ... | Asylum, Colney Hatch, London, N. |
| *SHAW, J. E., M.B., C.M. Ed. ... ... ... ... | 23 Caledonia Place, Clifton. |
| SHAW, JESSE, M.D.... ... ... ... ... ... ... | Fort Beaufort, Cape Colony. |
| SHEEN, A., M.D. St. And. ... ... ... ... ... | 23 Newport Road, Cardiff. |
| *SHEPPARD, E. J. ... ... ... ... ... ... ... | 14 Frederick Place, Clifton. |
| SHORTRIDGE, THOMAS WOOD, M.D. Brussels | Honiton. |
| SIMONS, C. E. G., M.B., C.M. Aberd. ... ... | The Hollies, Merthyr Tydvil. |
| SKELTON, HENRY, M.D. St. And. ... ... ... | Downend, Gloucestershire. |
| *SKERRITT, E. MARKHAM, M.D., B.S., B.A. Lond. | Richmond Hill, Clifton. |
| *SKINNER, S., M.B., C.M. Aberd. ... ... ... | Tranent Lawn, Clevedon. |
| *SMITH, G. MUNRO ... ... ... ... ... ... ... | 28 Alma Road, Clifton. |
| *SMITH, J. GREIG, M.B., C.M., M.A. Ab., F.R.S.E. | 16 Victoria Square, Clifton. |
| *SMITH, J. WESLEY, M.B., C.M. Aberd. ... ... | Boulevard, Weston-super-Mare. |
| SMITH, REV. REGINALD, M.A. Cantab. ... ... | Walpole St. Andrew's, Wisbeach. |
| *SMITH, R. SHINGLETON, M.D., B.Sc. Lond. ... | Clifton Park, Clifton. |
| SMITH, W. ALEXANDER, M.B., M.A. Oxon. ... | Newport, Essex. |

### LIST OF SUBSCRIBERS.

*Smith, W. A. L., M.A., M.B., B.C. Cantab. ... 8 Chamberlain Street, Wells.
Smyth, H. J. ... ... ... ... ... ... ... ... ... New Road, South Molton.
Society, Cardiff Medical (*Hon. Sec.*, Dr. D. R. Paterson).
Society, Columbian Medical ... ... ... ... 130 State Street, Chicago, U.S.A.
Society, Plymouth Medical (*Hon. Lib.*, C. E. R. Rendle).
Society, Torquay Medical (*Hon. Sec.*, G. Young Eales).
Solly, Reginald V., M.B., B.S. Lond. ... ... 40 Southernhay, Exeter.
Solly, S. E. ... ... ... ... ... ... ... ... Colorado Springs, Colorado, U. S. A.
Somer, James ... ... ... ... ... ... ... ... ... Broad Clyst.
Soutar, James Greig, M.B., C.M. Ed. ... ... Barnwood House, Gloucester.
Stamp, W. D. ... ... ... ... ... ... ... ... ... Ridgeway House, Plympton.
Statham, R. Whiteside ... ... ... ... ... Cheddar, Somersetshire.
*Steele, Charles, M.D. Dur. ... ... ... ... 17 Richmond Hill, Clifton.
Steele-Perkins, E. B.... ... ... ... ... ... 1 Hill's Buildings, Exeter.
*Stephenson, J. B., M.B., B.S. Dur. ... ... 19 Park Row, Bristol.
Stevens, W. H. ... ... ... ... ... ... ... ... Zetland Road, Bristol.
Stoddart, F. Wallis ... ... ... ... ... ... ... 1 Park Street, Bristol.
*Swain, James, M.D., M.S. Lond. ... ... ... 7 Buckingham Place, Clifton.
*Swayne, J. G., M.D. Lond. ... ... ... ... ... 74 Pembroke Road, Clifton.
*Swayne, S. H. ... ... ... ... ... ... ... ... ... 129 Pembroke Road, Clifton.
*Swayne, W. Carless, M.D. Lond. ... ... ... 8 Leicester Place, Clifton.
Swete, H. Lawton ... ... ... ... ... ... ... Fishguard, Pembrokeshire.
*Symes, J. O., M.D. Lond. ... ... ... ... ... 11 Richmond Hill, Clifton.
Symonds, H. P. ... ... ... ... ... ... ... ... 35 Beaumont Street, Oxford.
Symons, John ... ... ... ... ... ... ... ... ... Buriton House, Penzance.
Symons, W. H., M.D. Brux., D.P.H. Oxon. and Dur. ... ... ... ... ... ... ... ... Guildhall, Bath.

Taylor, C. Bell, M.D. Edin. ... ... ... ... 9 Park Row, Nottingham.

*Taylor, E. Claude, M.D. Lond. ... ... ... Children's Hospital, Bristol.

*Taylor, James ... ... ... ... ... ... ... ... ... 36 Alma Road, Clifton.

Thomas, A. Garrod, M.D., C.M. Ed. ... ... Clytha Park, Newport, Mon.

Thomas, J. Lynn ... ... ... ... ... ... ... ... 28 Charles Street, Cardiff.

Thomas, John T. ... ... ... ... ... ... ... ... Brynhyfryd, Chelston, Torquay.

Thomas, Rowland ... ... ... ... ... ... ... ... The Cwfr, St. Clears, Carmarthenshire.

Thomas, W. H.... ... ... ... ... ... ... ... ... Maesteg, Glamorganshire.

Thomson, Eustace B., M.D., C.M. Glasg. ... Albany Place, Plymouth.

*Tivy, W. J.... ... ... ... ... ... ... ... ... ... 8 Lansdown Place, Clifton.

Tolputt, Percy T.... ... ... ... ... ... ... ... 4 Downing Street, Farnham.

Tosswill, Louis H., M.B., B.A. Cantab.... ... 28 West Southernhay, Exeter.

Trevithick, Edgar, M.A., M.D. Cantab. ... 24 Promenade, Cheltenham.

Trotter, Leslie B., M.D., C.M. Ed. ... ... Coleford, Gloucestershire.

Tyley, R. P., M.D. St. And.... ... ... ... ... Wedmore.

Vachell, Herbert R., M.D., C.M. Aberd. ... 18 Newport Road, Cardiff.
Vereker, R. H. ... ... ... ... ... ... ... ... Eastleigh, Curry Rivel.

Wade, A. Law, M.D., B.A. Dub.... ... ... ... County Asylum, Wells, Somersetshire.

Wade, Charles H., M.A. Oxon. ... ... ... Stanfield, Torquay.

*Waldo, Henry, M.D., C.M. Aberd. ... ... ... 19 Pembroke Road, Clifton.

*Walker, Cyril H., M.B., B.A. Cantab. ... ... 3 Leicester Villas, Clifton.

Wallace, Rev. Canon, M.A. Oxon.... ... ... 3 Harley Place, Clifton.

*Wallace, John ... ... ... ... ... ... ... ... 1 Oriel Terrace, Weston-super-Mare.

Walsh, Leslie H. ... ... ... ... ... ... ... ... Royal United Hospital, Bath.

Ward, J. L. W.... ... ... ... ... ... ... ... ... Victoria Street, Merthyr Tydvil.

*Wathen, J. Hancocke ... ... ... ... ... ... ... 16 York Place, Clifton.

Watters, George T. B., M.D., C.M. Ed. ... Stonehouse, Gloucestershire.

| | |
|---|---|
| WAUGH, ALEXANDER ... ... ... ... ... ... ... | Midsomer Norton. |
| WEATHERLY, LIONEL A., M.D., C.M. Aberd. | Bailbrook House, Bath. |
| WEBBER, WILLIAM W. ... ... ... ... ... ... ... | Crewkerne. |
| WEDMORE, C. ERNEST, M.B., M.A. Cantab. ... | Stoke Bishop. |
| *WHICHER, A. H. ... ... ... ... ... ... ... | Midsomer Norton. |
| WHITBY, C. J., B.A., M.D. Cantab. ... ... ... | Limpley Stoke, Bath. |
| WICKHAM, W. ... ... ... ... ... ... ... ... ... | Hill House, Tetbury. |
| WIGAN, C. A., M.D. Dur. ... ... ... ... ... | Portishead. |
| WIGMORE, J., M.D. Roy. Univ. I. ... ... ... | 20 Green Park, Bath. |
| WILCOX, HENRY, M.B. Aberd. ... ... ... ... | Fleet, Hampshire. |
| WILDE, ROWLAND, M.B. Edin. ... ... ... ... | Park House, Weston-super-Mare. |
| *WILDING, JAMES, M.D. Dur. ... ... ... ... | 1 Apsley Villas, Kingsdown Parade, Bristol |
| WILLCOX, R. LEWIS ... ... ... ... ... ... | Warminster. |
| WILLETT, GEORGE G. D. ... ... ... ... ... | Keynsham, Somersetshire. |
| *WILLIAMS, E. C., M.B., B.C., B.A. Cantab. ... | 26 All Saints' Road, Clifton. |
| WILLIAMS, H. EGERTON ... ... ... ... ... | The Mount, Newport, Mon. |
| WILLIAMS, JOHN, M.D. ... ... ... ... ... ... | 20 Windsor Place, Cardiff. |
| *WILLIAMS, LIONEL H. ... ... ... ... ... ... | Thornbury, Gloucestershire. |
| *WILLIAMS, P. WATSON, M.D. Lond.... ... ... | 2 Lansdown Place, Clifton. |
| WILLIAMS, PETER ... ... ... ... ... ... ... | Ferryside, Carmarthenshire. |
| WILLIS, W. M.... ... ... ... ... ... ... ... | 8 Radnor Place, London, W. |
| *WINDSOR-AUBREY, H. W. ... ... ... ... ... | Coronation Road, Bristol. |
| WINTERBOTHAM, L. ... ... ... ... ... ... | Bays Hill, Cheltenham. |
| *WINTLE, COLSTON ... ... ... ... ... ... ... | 24 Gloucester Road, Bristol. |
| WOODFORD, E. RUSSELL, M.D., C.M. Ed. ... | 4 Prospect Terrace, Ventnor. |
| WOODHEAD, G. SIMS, M.D., C.M. Ed. ... ... | 1 Nightingale Lane, London, S.W. |
| WOODS, FRANK ... ... ... ... ... ... ... | Timsbury. |
| WOOLLCOMBE, WALTER L. ... ... ... ... ... | 16 Lockyer Street, Plymouth. |

*Subscribers are requested to send to the Editorial Secretary any change or alteration of address.*

---

### Bristol Medico-Chirurgical Journal.

| Terms for Advertisements. | £ | s. | d. |
|---|---|---|---|
| Page ... ... ... ... ... ... ... ... ... ... ... ... | 1 | 1 | 0 |
| Half Page ... ... ... ... ... ... ... ... ... ... | 0 | 12 | 6 |
| Quarter Page ... ... ... ... ... ... ... ... ... ... | 0 | 7 | 6 |

Insets (2 or 4 pages), 20/-

Upon orders given for four consecutive insertions, a discount of 25 per cent. will be allowed, if payment is made within one month from date of first insertion. Terms for special positions upon application.

*Orders to be sent to the Editorial Secretary*, B. ROGERS, M.D., *Clifton, Bristol.*

### PUBLICATIONS RECEIVED.

From P. Agnelli, Milan:

*Giornale della Reale Società Italiana d'Igiene.* Anno xix. Nos. 1—3. 1897.

From Baillière, Tindall and Cox, London:

*Diseases of Women.* By H. Macnaughton-Jones, M.D., M.Ch. Seventh Edition. 1897.
*Physiology. Students' Note-Book.* Part 1.—*Physiological Chemistry.* By Arthur J. Hall, B.A., M.B. 1897.

From John Bale & Sons, London:

*The Journal of Balneology and Climatology.* Vol. I. Part I. January, 1897.

From Friedr. Bayer & Co., Elberfeld:

*Clinical Excerpts.* Vol. I. Nos. 1, 2. January, February, 1897.

From L. Bergmann u. Comp., Vienna:

*Ueber den therapeutischen Werth des Tannalbins bei Darm- und Nierenerkrankungen.* Von Prof. Dr. G. Scognamiglio. 1897. [Reprint.]

From Burroughs, Wellcome & Co., London:

*The Year's Progress.* January 1, 1897.

From California Medical College, San Francisco:

*California Medical Journal.* December, 1896—February, 1897.

From Cassell and Company, Limited, London:

*The Practitioner.* Vol. LVI. 1896.

From W. P. Chew, London:

*The Happy Home.* Vol. V. No. 96. January 9, 1897.

From J. & A. Churchill, London:

*Fifty-four Consecutive Ovariotomies.* By A. C. Butler-Smythe. 1897.
*The Feeding of Infants.* By Edmund Cautley, M.D. 1897.
*The Operations of Surgery.* By W. H. A. Jacobson, M.Ch. Third Edition. 1897.
*On the History of the Respiration of Man.* By William Marcet, M.D., F.R.S. 1897.
*Moods: their Mental and Physical Character.* [F. Phillips.] 1897.

From Church Missionary Society, London:

*Mercy and Truth.* Vol. I. Nos. 1—3. January—March, 1897.

From William Clowes and Sons, London:

*Lecture on Psychiatric Institutions, the Austrian Law of Curatel, and separate Asylums for Drunkenness.* By Herr Director Schlangenhausen. 1896. [6 copies.]

From Dr. J. W. Cokenower, Des Moines, Iowa:

*Can Maternal Mental Emotions Produce Malformations, Deformities, or Birthmarks?* By J. W. Cokenower, A.M., M.D. 1894. [Reprint.]

From The F. A. Davis Co., Philadelphia:

*Autoscopy of the Larynx and Trachea.* By Alfred Kirstein, M.D. Translated by Max Thorner, A.M., M.D. 1897.

From O. H. Dreyer, St. Louis:

*Medical Review.* December 5, 12, 19, 26, 1896; January 2, 9, 16, 23; Feb. 6, 13, 20, 1897.

From Eduardo Dublan, Mexico:

*Boletin del Consejo superior de Salubridad.* Noviembre, Diciembre, 1896.

From Charles Wood Fassett, Saint Joseph, Mo.:

*American Medical Journalist.* Vol. I. No. I. January, 1897.

From G. Gounouilhou, Bordeaux:

*Résultats de l'Examen histologique de 64 Végétations adénoïdes.* Par le Dr. Brindel. 1896.
*Empyème du Sinus maxillaire chez les Enfants. Pathogénie et Traitement des Déviations et Éperons de la Cloison du Nez chez les jeunes Enfants.* Par le Dr. E. J. Moure. 1896.

From HALL AND ENGLISH, Birmingham:

*Transactions of the British Orthopædic Society.* Volume I. 1896.

From HUGH HOPKINS, Glasgow:

*The Medical Environment.* By D. CAMPBELL BLACK, M.D. [1896.]

From INGRAM BROTHERS, London:

*The English Illustrated Magazine.* January—March, 1897.

From KNOLL & Co., Ludwigshafen a/Rhein:

*Klinische Beobachtungen über Diuretin.* Von R. SIEVERS und T. W. TALLQVIST.—*Klinisches über Diuretin.* Von Dr. S. ASKANAZY—*Ueber Diuretica bei Herzkranken mit Compensationsstörungen.* Von Dr. med. THEODOR ZANGGER. 1895-96. [Reprint.]
*Erfahrungen über die Anwendung des Tannalbin in der Kinderpraxis.* Von Dr. J. G. REY 1897. [Reprint.]
*Tannalbin gegen Diarrhöen bei Erwachsenen und Kindern.*

From LAMBERT PHARMACAL Co., St. Louis:

*Listerine.* 1897.

From H. K. LEWIS, London:

*Rough Notes on Remedies.* By WM. MURRAY, M.D. Second Edition. 1897.
*What to Do in Cases of Poisoning.* By WILLIAM MURRELL, M.D. Eighth Edition. 1897.
*Compressed Air Illness.* By E. HUGH SNELL, M.D., B.Sc. 1896.
*On Deafness, Giddiness, and Noises in the Head.* By EDWARD WOAKES, M.D., assisted by CLAUD WOAKES. Fourth Edition. 1896.
*Dental Surgery.* By A. W. BARRETT, M.B. Third Edition. 1897.
*Ligaments.* By J. BLAND SUTTON. Second Edition. 1897.
*Diseases of the Eye.* By HENRY R. SWANZY, A.M., M.B. Sixth Edition. 1897.

From LUCIUS & BRUENING, Hoechst-on-Main:

*The Modern Therapeutics of the Farbwerke vorm: Meister Lucius & Bruening Medicinal Products.* 1897.

From WILLIAM F. MACK AND Co., Bristol:

*Bristol Medical Missionary Society. Twenty-fifth Annual Report.* 1896.

From MACMILLAN & Co., LTD., London:

*A System of Gynæcology.* By Many Writers. Edited by THOMAS CLIFFORD ALLBUTT, M.A., M.D., F.R.S., and W. S. PLAYFAIR, M.D. 1896.

From HORACE MARSHALL & SON, London:

*Travel.* Vol. I. Nos. 10, 11. February, March, 1897.

From "THE MEDICAL TIMES," LTD., London:

*The Medical Times and Hospital Gazette.* February 20, 1897.

From MITCHELL & HUGHES, London:

*The Middlesex Hospital Journal.* Vol. I. No. 1. January, 1897.

From THE NATIONAL TEMPERANCE LEAGUE PUBLICATION DEPOT, London:

*The Medical Pioneer.* Vol. 5. No. 2. February, 1897.

From Dr. CHARLES P. NOBLE, Philadelphia:

*The Diagnosis of Pregnancy during the first Three Months.* By CHARLES P. NOBLE, M.D. 1894. [Reprint.]
*Technique of Emptying the Uterus in Inevitable Abortion.* By CHARLES P. NOBLE, M.D. 1895. [Reprint.]
*Movable Kidney.* By CHARLES P. NOBLE, M.D. [N.D.] [Reprint.]

From P. A. NORSTEDT & SÖNER, Stockholm:

*Förhandlingar vid Första Nordiska Kongressen för Invärtes Medicin.* 1896.

From NOTES PUBLISHING Co., St. Louis:

*Notes on New Pharmaceutical Products.* Vol. VIII. No. 12. December, 1896.

From THE "OBSERVER" GENERAL STEAM PRINTING WORKS, Rochdale:

*The Dioptric and Ophthalmometric Review.* No. 3. October, 1896.

From YOUNG J. PENTLAND, Edinburgh:

*Treatment of Wounds, Abscesses and Ulcers.* By W. WATSON CHEYNE, M.B., F.R.S. Second Edition. 1897.

From JACOBO PEUSER, Buenos Ayres:

*El Cólera en la República Argentina.* Por el Doctor JOSÉ PENNA. 1897.

### PUBLICATIONS RECEIVED *(continued)*.

From THE REBMAN PUBLISHING COMPANY. LTD., London:

*Anomalies and Curiosities of Medicine.* By GEORGE M. GOULD, A.M., M.D., and WALTER L. PYLE, A.M., M.D. 1897.
*Archives of Clinical Skiagraphy.* No. 3. Vol. I. December, 1896.
*Injuries and Diseases of the Ear.* By MACLEOD YEARSLEY. 1897.
*Intestinal Intoxication in Infants.* By F. W. FORBES ROSS, M.D. 1897.
*Materia Medica, Therapeutics, and Pharmacology.* By GEORGE FRANK BUTLER, Ph.G., M.D. 1897.
*Sanatoria for Consumptives.* By Dr. ARTHUR VON JARUNTOWSKY. Translated by E. CLIFFORD BEALE, M.A., M.B. [N.D.]

From THE REPUBLICAN PRESS ASSOCIATION, Concord:

*Car Sanitation. Report of Committee, together with other Papers on that Subject.* 1895. [Reprint.]
*Car Sanitation. Report of Committee, together with other Papers on that Subject.* 1896. [Reprint.]

From JOHN MORGAN RICHARDS, London:

*Medical Reprints.* December, 1896—February, 1897.

From A. LA RIVIERE, London:

*Sight Testing for the G.P.* By F. DAVIDSON. [N.D.]

From Dr. AUGUST SCHACHNER, Louisville:

*An Improved Surgical Bed.* By AUGUST SCHACHNER, M.D. 1896. [Reprint.]

From A. SCHIFFER, Paris:

*The English & American Gazette.* 1st year. No. 12. February 6th, 1897.

From THE SCIENTIFIC PRESS, LIMITED, London:

*District Nursing.* By AMY HUGHES. 1897.
*Practical Points in Nursing.* By EMILY A. M. STONEY. [N.D.]
*The Menopause and its Disorders.* By A. D. LEITH NAPIER, M.D. 1897.

From SMITH, ELDER, & CO., London:

*Dr. Robert Watt.* By JAMES FINLAYSON, M.D. 1897.

From Dr. C. A. VEASEY, Philadelphia:

*Binasal Hemianopsia, with the Report of an Additional Case.* By CLARENCE A. VEASEY, A.M., M.D. 1897. [Reprint.]

From WILLIAMS AND NORGATE, London:

*Aetiologische Studien über Lepra.* Von Dr. EDWARD EHLERS. 1896.

*Die chirurgische Asepsis der Hände.* Von Dr. med. Wilh. Poten. 1897.

From JOHN WRIGHT & CO., Bristol:

*The Climate of Bournemouth in Relation to Disease, especially Phthisis.* By A. KINSEY-MORGAN, M.D. 1897.
*Elementary Bandaging and Surgical Dressing.* By WALTER PYE. Revised and in part Re-written by G. BELLINGHAM SMITH. Seventh Edition. 1896.
*Herbal Simples.* By W. T. FERNIE, M.D. Second Edition. 1897.

---

*Crown 8vo, with Illustrations,* 5/-

**PAST AND FUTURE:** With Addenda, being a Second Edition of **SATURN'S KINGDOM,** by CHARLES MOORE JESSOP, Member of the Royal College of Physicians, London, Deputy Surgeon-General H.M.'s Forces, is now ready.

"An infinite range of subjects, and much good sense brought to bear on each of them in turn. . . . . The deductions of the book are excellent, and it presents a wonderful amount of well-digested information in a particularly readable way."—*Daily Telegraph.*

**London: KEGAN PAUL, TRENCH, TRÜBNER & CO., LIMITED.**

*By the same Author, just published. Price 9d.*

**DRESS AND HEALTH:** An Appeal to Antiquity and Common Sense.

**London: ELLIOT STOCK, 62 Paternoster Row.**

Bristol Medico-Chirurgical Journal,

MARCH, 1897.

### EXCHANGE-LIST.

### AMERICA.

### ARGENTINE REPUBLIC.

*Weekly.*

*La Semana Médica.* Buenos Ayres: Cerrito, 475.

### CANADA.

*Monthly.*

*The Canadian Practitioner.* Toronto: The Practitioner Publishing Company.

*The Montreal Medical Journal.* Montreal: The Gazette Printing Company.

### MEXICO.

*Twice a month.*

*Gaceta Médica.* Mexico: Avenida 2 Oriente, 726.

### PERU.

*Twice a month.*

*La Crónica Médica.* Lima: 96 Calle y Plaza de San Pedro.

### UNITED STATES.

*Weekly.*

*Boston Medical and Surgical Journal.* Boston: Damrell & Upham.

*Medical Record.* New York: William Wood & Co.

*The Cincinnati Lancet-Clinic.* Cincinnati: 317 West 7th Street.

*The Journal of the American Medical Association.* Chicago: 61 Market Street.

*The Medical and Surgical Reporter.* Philadelphia: The Butler Publishing Company.

*The Medical News.* New York: Lea Brothers & Co.

*The New York Medical Journal.* New York: D. Appleton & Co.

*Fortnightly.*

*The American Practitioner and News.* Louisville: John P. Morton and Company.

*Twice a month.*

*Pediatrics.* New York: Van Publishing Co.

*The Medical Age.* Detroit: George S. Davis.

*Monthly.*

*Archives of Pediatrics.* New York: E. B. Treat.

*Buffalo Medical Journal.* Buffalo: A. T. Brown Printing House.

*Bulletin of the Johns Hopkins Hospital.* Baltimore: The Johns Hopkins Press.

*International Medical Magazine.* Philadelphia: International Medical Magazine Company.

*Journal of Cutaneous and Genito-Urinary Diseases.* New York: The Physicians' Publishing Company.

*Occidental Medical Times.* Sacramento: D. Johnston & Co.

*The American Gynæcological and Obstetrical Journal.* New York: 91 Madison Avenue.

*The American Journal of Obstetrics and Diseases of Women and Children.* New York: William Wood & Co.

*The American Journal of Ophthalmology.* St. Louis: 3036 Locust Street.

*The American Journal of the Medical Sciences.* Philadelphia: Lea Brothers & Co.

*The Brooklyn Medical Journal.* Brooklyn: 260 Hancock Street.

*The Journal of Nervous and Mental Disease.* New York: 25 West 45th Street.

*The Laryngoscope.* St. Louis: 707 Olive Street.

*The Post-Graduate.* New York: 2nd Avenue and 20th Street.

*The Saint Louis Medical and Surgical Journal.* Saint Louis: The Commercial Printing Company.

*The Therapeutic Gazette.* Detroit: George S. Davis.

*The Universal Medical Journal.* Philadelphia: The F. A. Davis Co.

*University Medical Magazine.* Philadelphia: University of Pennsylvania Press.

*Bi-monthly.*

*The Journal of Experimental Medicine.* New York: D. Appleton and Company.

*Quarterly.*

*Mathews' Quarterly Journal of Rectal and Gastro-Intestinal Diseases.* Louisville: John P. Morton & Company.

*The Alienist and Neurologist.* St. Louis: Hughes & Company.

*Yearly.*

*Annual of the Universal Medical Sciences.* Philadelphia: The F. A. Davis Company.

*The American Year-Book of Medicine and Surgery.* Philadelphia: W. B. Saunders.

*Transactions of the American Association of Obstetricians and Gynecologists.* Philadelphia: William J. Dornan.

*Transactions of the American Dermatological Association.* New York: Geo. L. Goodman & Co.

*Transactions of the American Gynecological Society.* Philadelphia: William J. Dornan.

*Transactions of the American Laryngological Association.* New York: D. Appleton and Company.

*Transactions of the American Ophthalmological Society.* Hartford: 26 Pratt Street.

*Transactions of the American Pediatric Society.* New York: Rooney & Otten Printing Co.

*Transactions of the American Surgical Association.* Philadelphia: William J. Dornan.

*Transactions of the Association of American Physicians.* Philadelphia: William J. Dornan.

*Transactions of the College of Physicians of Philadelphia.* Philadelphia: William J. Dornan.

*Transactions of the Colorado State Medical Society* Denver: A. J. Luddit.

*Transactions of the Iowa State Medical Society.* Des Moines: The Kenyon Printing and Mfg. Co.

*Transactions of the Medical Association of the State of Alabama.* Montgomery: Brown Printng Co.

*Transactions of the Medical Society of New Jersey.* Newark: L. J. Hardham.

*Transactions of the Medical Society of the State of New York.* Philadelphia: William J. Dornan.

*Transactions of the Medical Society of Virginia.* Richmond: J. W. Fergusson & Son.

*Transactions of the Michigan State Medical Society.* Grand Rapids: Seymour & Muir Printing Co.

*Transactions of the New Hampshire Medical Society.* Concord: Republican Press Association.

*Transactions of the New York Academy of Medicine.* New York: Stettiner, Lambert & Co.

*Transactions of the Ohio State Medical Society.* Toledo: The Blade Printing and Paper Co.

*Transactions of the South Carolina Medical Association.* Charleston: Walker, Evans & Cogswell Co.

*Transactions of the Texas State Medical Association.* Galveston: Knapp Brothers.

### ASIA.

### CHINA.

*Quarterly.*

*The China Medical Missionary Journal.* Shanghai: The American Presbyterian Mission Press.

### INDIA.

*Monthly.*

*The Indian Medico-Chirurgical Review.* Bombay: N. K. Rao & Co.

### JAPAN.

*Monthly.*

*The Sei-I-Kwai Medical Journal.* Tokio: 6 Shin Sakana Chō, Kyōbashi Ku.

### AUSTRALASIA.

### AUSTRALIA.

*Monthly.*

*Intercolonial Medical Journal of Australasia.* Melbourne: Stillwell and Co.

*The Australasian Medical Gazette.* Sydney: 121 Bathurst Street.

### EUROPE.

### AUSTRIA.

*Weekly.*

*Wiener klinische Wochenschrift.* Vienna: Wilhelm Braumüller.

### BELGIUM.

*Twice a month.*

*Journal de Neurologie & d'Hypnologie.* Brussels: A. Gossé & Ce.

*Monthly.*

*Bulletin de l'Académie royale de Médecine de Belgique.* Brussels: F. Hayez.

### BRITISH ISLES.

*Weekly.*

*British Medical Journal.* London: 429 Strand.

*Medical Press and Circular.* London: A. A. Tindall.

*The Clinical Journal.* London: E. Knight.

*The Hospital.* London: The Scientific Press (Limited).

*The Lancet.* London: 423 Strand.

*The Sanitary Record.* London: The Sanitary Publishing Co., Ltd.

*Monthly.*

*Edinburgh Medical Journal.* Edinburgh: Young J. Pentland.

*The Birmingham Medical Review.* Birmingham: Hall & English.

*The British Journal of Dermatology.* London: H. K. Lewis.

*The Dublin Journal of Medical Science.* Dublin: Fannin & Co., Ltd.

*The Journal of Laryngology, Rhinology, and Otology.* London: The Rebman Publishing Company, Limited.

*The Medical Chronicle.* Manchester: John Heywood.

*The Practitioner.* London: Cassell & Company, Limited.

*The Scottish Medical and Surgical Journal.* Edinburgh: William F. Clay.

*Transactions of the Odontological Society of Great Britain.* London: John Bale & Sons.

*Quarterly.*

*Archives of Surgery.* London: West, Newman & Co.

*The British Gynæcological Journal.* London: John Bale & Sons.

*The Northumberland and Durham Medical Journal.* Newcastle-upon-Tyne: The "Daily Journal" Office.

*The Quarterly Medical Journal.* Sheffield: Pawson and Brailsford.

*The West London Medical Journal.* London: John Bale & Sons.

*Half-yearly.*

*The Liverpool Medico-Chirurgical Journal.* Liverpool: Medical Institution.

*The Retrospect of Medicine.* London: Simpkin & Co. Limited.

*Yearly.*

*Clinical Society's Transactions.* London: Longmans, Green, and Co.

*Guy's Hospital Reports.* London: J. & A. Churchill

*King's College Hospital Reports.* London: Adlard & Son.

*Medico-Chirurgical Transactions.* London: Longmans, Green, and Co.

*Obstetrical Transactions.* London: Longmans, Green, and Co.

*Ophthalmological Transactions.* London: J. & A. Churchill.

*Saint Bartholomew's Hospital Reports.* London: Smith, Elder & Co.

*St. Thomas's Hospital Reports.* London: J. & A. Churchill.

*The Medical Annual.* Bristol: John Wright & Co.

*The Medical Society's Transactions.* London: Harrison & Sons.

*The Transactions of the Edinburgh Obstetrical Society.* Edinburgh: Oliver and Boyd.

*The Westminster Hospital Reports.* London: J. & A. Churchill.

*The Year-Book of Treatment.* London: Cassell & Company, Limited.

*Transactions of the Dermatological Society of Great Britain and Ireland.* London: H. K. Lewis.

*Transactions of the Epidemiological Society of London.* London: Shaw and Sons.

*Transactions of the Royal Academy of Medicine in Ireland.* Dublin: Fannin & Co., Ltd.

*Year-Book of Scientific and Learned Societies.* London: C. Griffin & Co., Ltd.

BRITISH ISLES (*continued*).

*Occasionally.*

*The International Directory of Second-hand Booksellers.* Rochdale: James Clegg.

DENMARK.

*Weekly.*

*Hospitalstidende.* Copenhagen: Jacob Lund.

FRANCE.

*Three times a week.*

*Gazette des Hôpitaux.* Paris: 4 Rue de l'Odéon.

*Weekly.*

*Bulletin de l'Académie de Médecine.* Paris: Masson et Cie.

*Gazette hebdomadaire des Sciences médicales de Bordeaux.* Bordeaux: P. Cassignol.

*Gazette des Hôpitaux de Toulouse.* Toulouse: 3 Avenue Lafayette.

*La France médicale.* Paris: 9 Rue des Pyramides.

*La Revue médicale.* Paris: 69 Boulevard Saint-Michel.

*Le Progrès médical.* Paris: 14 Rue des Carmes.

*Revue hebdomadaire de Laryngologie, d'Otologie et de Rhinologie.* Paris: Octave Doin.

*Fortnightly.*

*Le Nord médical.* Lille: 209 Rue Léon-Gambetta.

*Twice a month.*

*Gazette de Gynécologie.* Paris: 12 Rue de Châteaudun.

*Revue de Thérapeutique médico-chirurgicale.* Paris: 8 Rue de Castellane.

*Monthly.*

*Archives cliniques de Bordeaux.* Bordeaux: 8 Rue de Cheverus.

*Archives de Neurologie.* Paris: 14 Rue des Carmes.

*Revue générale d'Ophtalmologie.* Paris: Masson et Cie.

*Revue des Instruments de Chirurgie.* Paris: 4 Rue Royer-Collard.

*Revue médico-chirurgicale des Maladies des Femmes.* Paris: 45 Boulevard Malesherbes.

*Revue pratique d'Obstétrique et de Gynécologie.* Paris: 47 Boulevard Haussmann.

GERMANY.

*Weekly.*

*Centralblatt für Chirurgie.* Leipzig: Breitkopf & Härtel.

*Centralblatt für Gynäkologie.* Leipzig: Breitkopf & Härtel.

*Centralblatt für innere Medicin.* Leipzig Breitkopf & Härtel.

*Münchener medicinische Wochenschrift.* Munich: J. F. Lehmann.

*Monthly*

*Zeitschrift für Krankenpflege.* Berlin: H. Kornfeld.

GREECE.

*Twice a month.*

*Γαληνὸς.* Athens: 3 Rue de Marseille.

*'Ιατρικὴ 'Επιθεώρησις.* Athens: 40 Rue Zinonos.

HOLLAND.

*Weekly.*

*Nederlandsch Tijdschrift voor Geneeskunde.* Amsterdam: F. van Rossen.

ITALY.

*Weekly.*

*Gazzetta Medica Lombarda.* Milan: 40 Via Senato.

*Twice a month.*

*Giornale Internazionale delle Scienze Mediche.* Naples: Detken & Rocholl.

*Monthly.*

*Annali di Ostetricia e Ginecologia.* Milan: L. F. Cogliati.

*Bi-monthly.*

*Archivio di Ortopedia.* Milan: Pietro Agnelli.

NORWAY.

*Monthly.*

*Norsk Magazin for Lægevidenskaben.* Christiania: Steenske Bogtrykkeri.

PORTUGAL.

*Weekly.*

*A Medicina Contemporanea.* Lisbon: José Antonio Rodrigues.

RUSSIA.

*Weekly.*

*Medycyna.* Warsaw: 38 Rue de Varenne.

*St. Petersburger Medicinische Wochenschrift.* St. Petersburg: Carl Ricker.

*Vrach.* St. Petersburg: C. Ricker.

*Monthly.*

*Finska Läkaresällskapets Handlingar.* Helsingfors: Helsingfors Central-Tryckeri.

SPAIN.

*Weekly.*

*El Siglo Médico.* Madrid: Magdalena, 36.

SWEDEN

*Bi-monthly.*

*Nordiskt Medicinskt Arkiv.* Stockholm: P. A. Norstedt & Söner.

TURKEY.

*Twice a month.*

*Gazette médicale d'Orient.* Constantinople: A. Christidis.

